# Merremia emarginata Extract Potentiates the Inhibition of Human Colon Cancer Cells (HT-29) via the Modulation of Caspase-3/Bcl-2-Mediated Pathways

**DOI:** 10.7759/cureus.56300

**Published:** 2024-03-16

**Authors:** Andrew Benedict, Vasugi Suresh, Muthamizh Selvamani, Selvaraj Jayaraman, Mohammed Asif Hussein

**Affiliations:** 1 Physiology, Saveetha Dental College and Hospitals, Saveetha Institute of Medical and Technical Science, Saveetha University, Chennai, IND; 2 Biochemistry, Saveetha Dental College and Hospitals, Saveetha Institute of Medical and Technical Science, Saveetha University, Chennai, IND

**Keywords:** elika-jemudu, merremia emarginata, bcl-2 and bcl-xl, ethanolic extract, cancer, mtt assay, herbal medicine, apoptosis, cell viability test, solvent extraction method

## Abstract

Background

This study investigates *Merremia emarginata*'s curative effectiveness against colon cancer cells. *M. emarginata*, often known as Elika jemudu, is a Convolvulaceae family plant. The inhibitory ability of anticancer herbal extracts against cancer cell growth and mediators is tested.

Aim

This study aims to evaluate the potent anticancer activity of *M. emarginata *against colon cancer cell line (HT-29).

Materials and methods

*M. emarginata* leaves were gathered and processed using solvent extraction. Anticancer activity on colon cancer cells was measured using the 3-(4,5-dimethylthiazol-2-yl)-2,5-diphenyltetrazolium bromide (MTT) test and cysteine aspartic acid protease-3 (caspase 3), B-cell lymphoma 2 (Bcl-2), and B-cell lymphoma-extra large (Bcl-xL) mRNA expressions. The data was reported as the mean ± SD of three separate experiments done in triplicate. The statistical analysis was carried out using one-way analysis of variance (ANOVA), with a p-value less than 0.05 indicating statistical significance.

Results

The cell viability test showed a gradual decrease in cell growth and proliferation as the concentration increased. The ethanolic extract of *M. emarginata* was found to be cytotoxic against colon caller cell lines. The extract was able to induce apoptosis of cancer as revealed by Bcl-2, Bcl-xL, and caspase-3 (p<0.05 and p<0.001) signaling pathways.

Conclusion

*M. emarginata* extracts showed good anticancer activity against colon cancer cell lines. Further work is required to establish and identify the chemical constituent responsible for its anticancer activity.

## Introduction

A pathological collapse in the mechanisms governing cell division, proliferation, and death is what defines cancer [1]. The third most prevalent kind of malignant tumor is colon cancer which affects millions of people worldwide [2]. Colon cancer accounted for about 9.7% of cancer diagnoses and 8.5% of cancer-related deaths worldwide in 2012. To address the cancer mortality burden in India, comprehensive solutions are required, including prevention, early detection, diagnosis, treatment, palliative care, and support services. Cancer mortality reduction efforts include promoting healthy behaviors, implementing evidence-based screening programs, improving access to quality cancer care, advancing research and innovation, and addressing social determinants of health in order to achieve health equity and reduce cancer disparities. The age of those who develop colorectal cancer and their risk are positively correlated. The fact that lifestyle variations exist across the globe demonstrates how lifestyle changes can genuinely impact the incidence of colorectal cancer. Many countries have seen a gradual but noticeable improvement in the prognosis of patients with colorectal cancer in the past few decades [3]. The best way to lower cancer-related morbidity and death rates is to use anticancer drugs to slow down the cancer's genesis. This means that safer substances, particularly those derived from natural sources, must be thoroughly investigated for their ability to fight cancer [4]. One of the primary advantages of working in vitro is that it allows for a high degree of system simplicity, which frees up the researcher to focus on fewer constituents. It is generally agreed upon in the literature as a result of in vitro cell culture experiments that some polyunsaturated fatty acids (PUFAs) selectively kill or inhibit the growth of tumor cells while having little to no effect on normal cells [5]. Because of their therapeutic properties, medicinal plants have been used for many years to treat a wide range of diseases [6].

Herbal medicine has a variety of antioxidant chemicals that combat free radicals and stop lipid peroxidation. Flavonoids and phenol are examples of secondary metabolites found in plants. These compounds can neutralize free radicals and have antioxidant qualities, which help prevent diseases like cancer [7]. Worldwide, traditional medicines, or TRM, are used extensively. A variety of therapeutic modalities, an empirical treatment approach, a materia medica, a theoretical foundation, and a training tradition characterize the majority of TRM systems. On TRM, a great deal of scientific research has been done and published [8]. Herbal medicines are a synthesis of several historical generations' therapeutic experiences mixed with the medical practices of indigenous systems, which offer helpful instructions for choosing, preparing, and using herbal formulations for the management, control, and treatment of a variety of ailments. It has been reported that plant-based medications can effectively treat a variety of infectious diseases, including AIDS, cancer, diabetes, jaundice, tuberculosis (TB), hypertension, and skin conditions. Ancient civilizations such as Egypt, China, India, and South America continue to treat these kinds of illnesses with a variety of plant-based cures. The World Health Organisation (WHO) estimates that 60% of people worldwide use herbal medicine and that in developing nations, 80% of people rely almost exclusively on it for their basic medical needs. Additionally, the necessity of further research into creating herbal medications as contemporary therapeutic agents is discussed [9].

The perennial herb *Merremia emarginata* Burm. F. (Convolvulaceae) has many branches, making it a creeper. The common name for it is* Ipomoea reniformis*. Known by most as the morning glory family, the Convolvulaceae is made up of roughly 60 genera and likely 1650 species. It's an uncultivated food crop that the underprivileged in India use as a green-leaf vegetable. It also roots as a creeping perennial herb at the nodes. The leaves are ovate-cordate or reniformis, simple, and have long stalks. The fruits resemble sub-globose capsules with two to four light brown, glabrous seeds, and the flowers are yellow and auxiliary, with one to three flowers on very short peduncles [10]. 

*Ipomoea reniformis* has been claimed to be helpful for a variety of conditions, including kidney diseases, rheumatism, inflammation, nose problems, fever from enlarged liver, headaches, neuralgia, and coughs. The juice of the leaves serves as a purgative, the powdered leaves are used as snuff during epileptic seizures, and the root is applied topically to treat gum and eye diseases. The plant has been found to possess nephroprotective, antipyretic, antibacterial, antidiabetic, antioxidant, anticancer, anti-inflammatory, anti-arthritic, and analgesic properties. Gas chromatography-mass spectrometry (GC-MS) analysis, in vitro antioxidant and antimicrobial activities, antioxidant, cytotoxic, and anti-inflammatory properties, hepatoprotective, anthelmintic, and antihypertensive properties have additionally been published in a few pharmacognostic studies [11].

Apoptosis is a type of prearranged cell death that is brought on by cell shrinkage. Gene mutations can occasionally lead to an increase in apoptosis, which ultimately results in cell death. Numerous research has been conducted on the induction of apoptosis using medicinal plants. However, given its potential therapeutic application, more research is required [12]. It is well known that immune activation brought on by a persistent infection or inflammation increases the risk of cancer. The immune system plays a significant role in the development and treatment of cancer. The tumor microenvironment (TME) is continuously shaped by neoplastic cells during tumor development, and TME becomes increasingly immunosuppressive [13].

The two most common medical sectors where novel medications are derived from medicinal plants are those of infectious diseases and cancer. With so many adverse effects from cancer treatments, the usage of herbal remedies that can either suppress or kill cancer cells through their antioxidant capacities is becoming more and more popular. There have been reports of the therapeutic use of many *M. emarginata* components for diuretics and neuralgia. Free radicals can be scavenged by *M. emarginata* extract, which makes it a notable natural antioxidant source. Toxicity studies indicate that most *Merremia* species are safe for human usage, but not for long-term, chronic administration [14].

Evaluating *M. emarginata* potent anticancer activity against colon cancer cells is the study's primary goal.

## Materials and methods

Chemicals

Gibco, Canada supplied trypsin-disodium ethylenediaminetetraacetic acid (EDTA), fetal bovine serum (FBS), antibiotics-antimycotics, Dulbecco's modified Eagle's medium (DMEM), and phosphate-buffered saline (PBS). The real-time polymerase chain reaction (PCR) kit (MESA Green) and 5,5,6,6-tetrachloro-1,1,3,3-tetra ethyl benzimidazole carbocyanine iodide (JC-1) were acquired from Invitrogen, USA. Every chemical that was utilized was extra pure and of analytical grade.

Extract preparation

*Merremia emarginata* powder was soxhlet extracted with 70% ethanol. The extract was then filtered through Whatman No. 1 filter paper (Whatman Plc, Maidstone, UK), and the solvent evaporated at reduced pressure using a rotary evaporator apparatus to produce a viscous mass that was stored at 4°C until use.

Procurement and culture of human colon cancer cell lines (HT-29)

The National Centre for Cell Science (NCCS), Pune, India is the source of the HT-29 cell line, which was cultured following the guidelines, supplied, and maintained at ambient temperature.

Assessment of cell viability by MTT

In 96-well plates, colon cancer cells (HT-29) were seeded at a density of 5x10^5^ cells/well and left to adhere to the well overnight. Following incubation, cultured cells were stimulated in triplicate with varying concentrations of *M. emarginata *extract and incubated for 24 hours at 37˚C in an incubator with 5% humidified CO_2_. Each well was then filled with 3-(4,5-dimethylthiazol-2-yl)-2,5-diphenyltetrazolium bromide (MTT), and the incubation was kept at 37˚C for an additional 4 hours. The cells were resuspended in 200 µl dimethylsulfoxide (DMSO) to dissolve the formazan formed from MTT. The optical density (OD) of the solution was measured with a spectrometer set to 570 nm. The inhibitory rate of cell growth was calculated using the equation [15]:

 % Growth inhibition = (1 - OD extract treated)/OD negative control x 100

Gene expression analysis by real-time PCR

The present material was adopted from previously reported literature [14]. Real-time PCR was used to analyze mRNA expression levels. The total RNA was extracted using TRI Reagent® (Sigma). Total RNA (2 μg) from each sample was reverse transcribed using a commercial SuperScript® III First-Strand cDNA Synthesis kit (Invitrogen, USA) following the manufacturer's procedure. The data was processed using the comparative C(T) method, and the fold change was determined using the 2-C(T) method reported by Schmittgen and Livak (2008) with CFX Manager version 2.1 (Bio-Rad, USA). Data were presented as the mean ± SD of three independent experiments conducted in triplicate. One-way analysis of variance (ANOVA) was used for statistical analysis, with p-values <0.05 indicating statistical significance.

Statistical analysis

The means ± SD of three distinct experiments conducted in triplicate were used to express the data. The one-way ANOVA was used for the statistical analysis, and a result was deemed statistically significant if it had a p-value of less than 0.05.

## Results

Effects of *M. emarginata* on HT-29 cell viability

**Figure 1:**
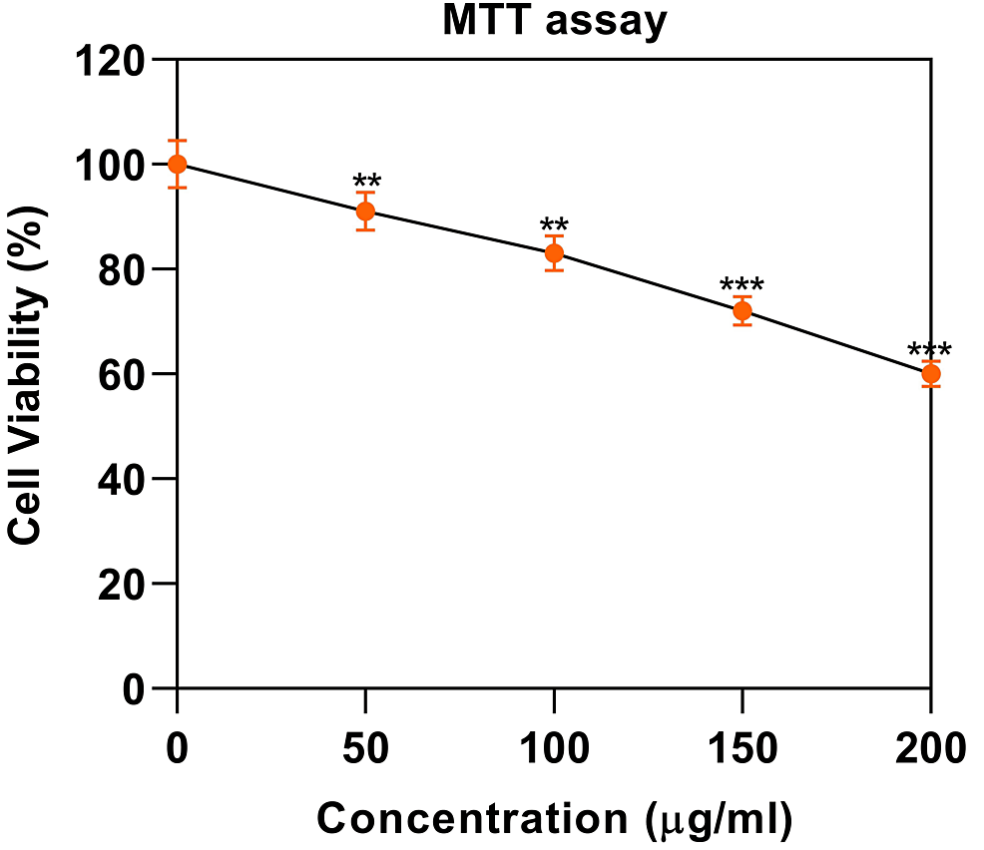
The effect of Merremia emarginata on cell viability in HT-29 cells by MTT method HT-29 cells were treated with different concentrations (0, 50, 100, 150, and 200 µg/ml) of plant extract (*M. emarginata*). The results are expressed in the percentage of viable cells in response to drug treatment. Statistical significance p<0.05**, p<0.001*** MTT: 3-(4,5-dimethylthiazol-2-yl)-2,5-diphenyltetrazolium bromide

The MTT assay is a common colorimetric assay used to measure cell viability and proliferation, often employed in anticancer studies to assess the cytotoxic effects of compounds on cancer cells. In the present study, we assayed the role of *M. emarginata *extract on HT-29 cells to check whether it can inhibit the growth of the HT-29 cells. Our study showed that* M. emarginata *reduced cancer cell progression in a dose-dependent fashion 0, 50, 100, 150, and 200 µg/mL concentration with respective inhibition was found to be 100%, 91%, 83%, 75%, and 58 %, respectively (Figure 1).

Effects of* M. emarginata *on the mRNA expression of apoptosis signaling molecules in HT-29 cells 

**Figure 2:**
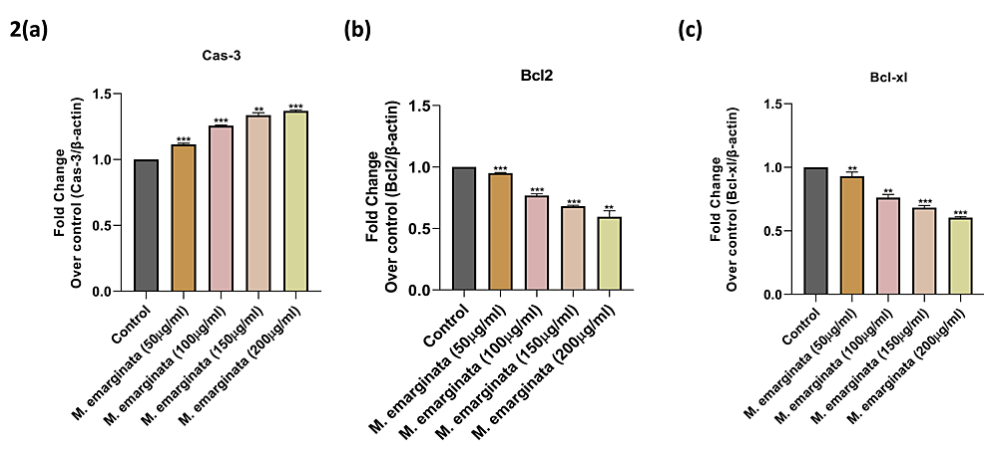
Effects of Merremia emarginata on the mRNA expression of apoptotic target signaling molecules (caspase 3, Bcl-2, and Bcl-xL) in human HT-29 cells mRNA levels of caspase 3, Bcl-2, and Bcl-xL were analyzed by real-time PCR method using gene-specific primers. Results are expressed in fold change over control (untreated cells). Statistical significance p<0.05**, p<0.001*** Cas-3: Caspase 3; PCR: Polymerase chain reaction

In the context of colon cancer, the proteins cysteine aspartic acid protease-3 (caspase 3), B-cell lymphoma 2 (Bcl-2), and B-cell lymphoma-extra large (Bcl-xL) are of particular interest due to their roles in apoptosis (programmed cell death) and their potential implications in cancer progression, including colon cancer. Hence, we measured the levels of caspase-3, Bcl-2, and Bcl-xL mRNA to check whether *M. emarginata *inhibits HT-29 cell proliferation via the regulation of apoptotic signaling mechanisms. mRNA levels were analyzed by real-time PCR methods and expressed in terms of fold change expression in comparison with untreated and treated cells. As shown in Figure 2a, caspase-3 expression was found to be 0.2, 0.4, and 0.5 fold reduced in 50, 100, 150, and 200 µg, respectively, in response to plant extract treatment (p<0.05, p<0). Anti-apoptotic molecules such as Bcl-2 and Bcl-xL expression were significantly reduced in extract-treated cells in a dose-dependent fashion (from 0.2, 0.4, and 0.5 fold) as depicted in Figure 2b and Figure 2c. This study strongly suggests that *M. emarginata* has a significant role in controlling HT-29 cell proliferation via the caspase-3, Bcl-2, and Bcl-xL mediated mechanisms.

## Discussion

The statistical analysis of the data in Figure 1 suggests that *M. emarginata *extracts had cytotoxic effects on the HT-29 cell in a dose-dependent manner. The MTT assay evaluates mitochondrial activity by causing living cells to convert MTT to formazan crystals. This is because the total mitochondrial activity of most cells is associated with the number of viable cells [16].

The highest amount of cancer cell growth inhibition was observed at 200 µg/ml of *M. emarginata *leaf extract, with a percentage of 50% cell growth inhibition in MTT assay. Cell viability dramatically drops as leaf extract concentration rises from 50 µg/ml to 200 µg/ml. It has been discovered that the anticancer activity of secondary plant metabolites, such as flavonoids and phenols, which are antioxidants, is specific and has the potential to treat and prevent cancer apoptosis on human cancer cell lines. The MTT assay of various plant fractions of *M. emarginata *Burm. F. demonstrates that all fractions exhibit anticancer activity. Methanol and ethyl acetate exhibit strong activity, while ethyl acetate demonstrates exceptionally strong anticancer activity. The ethyl acetate fraction's IC50 values against HeLa and MCF cells are 51.57 µg/ml and 39.6 µg/ml, respectively. This fraction was found to have specific anticancer activity and was able to cause apoptosis in human cancer cell lines [17]. It was found that *M. emarginata* exhibited cytotoxic activity in different sections; however, the only section that demonstrated activity was the ethyl acetate fraction, with an IC50 value less than 200 µg/ml. Upon evaluation of the remaining fractions, an IC50 value exceeding 200 µg/ml was considered inactive [18].

The statistical analysis of the data in Figures 2b-2c suggests that when *M. emarginata* was treated with 200 µg/ml, there was a noticeable decrease in Bcl-2 mRNA. Also, the concentration of Bcl-xL mRNA was significantly lower at 100 µg/ml and 200 µg/ml. It was discovered that *M. emarginata's* ethanolic extract was cytotoxic to colon cancer cell lines. The extract might induce apoptosis in cancer cells, as shown by the Bcl-2 and Bcl class signaling pathways. Finding and identifying the chemical component that gives it its anticancer capabilities will require additional investigation. *Bolbostemma paniculatum* (Maxim.) Franquet (Cucurbitaceae) tubers ethanol extracts revealed the presence of a novel minor component, Tubeimoside V (1). Tubeimoside V (1) was found by Cheng et al. to cause apoptosis in human glioma U87MG cells by upregulating the expression of Bcl-2-associated protein x (Bax) protein and decreasing that of Bcl-2 protein [19]. Furthermore, Figure 2a reveals that caspase activation is increased by caspase-3 cleavage of Bcl-2 as a component of a vicious feedback loop that destroys the cell. Bcl-2 family members have been investigated as therapeutic targets in a large variety of solid tumors as well as in chondrosarcoma. The *M. emarginata* extracts were tested for their ability to prevent tumor necrosis factor alpha (TNF α) from being secreted by lipopolysaccharide (LPS)-induced THP-1 cells. Out of the three extracts, the ethyl acetate extract showed good activity against the production of TNF α, with an IC50 of 5.9 µg/ml, which was followed by IC. However, in comparison to other extracts, the hexane and methanol extract exhibited only modest anti-inflammatory and anticancer effects. LPS is considered a strong stimulant of the inflammatory response in monocytes, triggering the activation of mitogen-activated protein (MAP) kinase enzymes, which in turn increases the production of TNF α13. TNF α plays a crucial role in controlling inflammatory illnesses like atherosclerosis, psoriasis, asthma, rheumatoid arthritis, and Crohn's disease. Three extracts were studied in vitro; in particular, the *M. emarginata* ethyl acetate extract showed promising anti-inflammatory and anticancer effects [20]. However, the cytotoxicity of the ethyl acetate is too high, and it may result in damaging the other normal cells. So compared with other extraction reagents like ethyl acetate, methanol, and hexane, the ethanolic extract is less harmful to normal cells.

Limitations

To advance this field of research, rigorous experimental design, detailed characterization, and evaluation of potential applications and safety concerns are essential. Collaborating with specialists in microbiology, cytology, and oncology can help overcome hurdles. The current work focuses on in vitro assessments. To increase the credibility of *M. emarginata *leaf extracts, in vivo research and clinical studies are needed to validate their usefulness and safety. The chemical structure of the medicine can also be examined and evaluated.

## Conclusions

The ethanolic extract of *M. emarginata *exhibits anticancer properties in HT-29 cells by modulating the expression of apoptosis signaling molecules in human colon cancer cells. This study provides in vitro experimental evidence that *M. emarginata* could be considered a therapeutic natural drug for the treatment of colon cancer. Further studies on protein expression of downstream signaling molecules of apoptosis pathways need to be considered.
